# Biobanking of gynecologic cancer biospecimens: Development, quality control, and translational applications

**DOI:** 10.1371/journal.pone.0345861

**Published:** 2026-03-31

**Authors:** Jue Young Kim, Yoo-Kyung Lee, Ha-Yeon Shin, Yoon Joo Kim, Mi Ae Jeon, Wookyeom Yang, Anna Jun, Kyungmin Kim, Hanbyoul Cho, Min-A Kim, Jae-Hoon Kim

**Affiliations:** 1 Department of Obstetrics and Gynecology, Gangnam Severance Hospital, Yonsei University College of Medicine, Seoul, Republic of Korea; 2 Department of Anti-cancer evaluation team, R&D Institute, ORGANOIDSCIENCES Ltd., Seongnam-si, Republic of Korea; Teikyo University, School of Medicine, JAPAN

## Abstract

**Introduction:**

This study presents a nationwide infrastructure for the collection and utilization of gynecologic cancer biospecimens, established through the Korea Biobank Project. We comprehensively describe the biobanking strategy, quality control protocols, and development of secondary resources to support future translational and discovery-based research.

**Methods:**

We established a gynecologic cancer biobank within the Korea Biobank Project (KBP) through a multi-institutional consortium. Biospecimens, including blood, tumor tissue, urine, and ascites, were collected from 294 patients with endometrial, cervical, or ovarian cancers. Pre-analytical variables were documented using the Standard PREanalytical Code (SPREC), and all samples were tracked with 2D barcodes. Secondary resources were developed, including whole-genome sequencing (WGS) datasets, immortalized human ovarian surface epithelial (IHOSE) cell lines, patient-derived xenografts (PDX), tumor organoids, and tissue microarrays (TMAs).

**Results:**

A total of 6,168 biospecimens were archived. WGS was performed on 386 cancer samples, including 172 paired tumor–normal sets. Four IHOSE cell lines were authenticated and validated for stability, 14 PDX models retained histological fidelity across passages, and patient-derived ovarian cancer organoids demonstrated drug sensitivity consistent with clinical response patterns. TMAs were constructed from 519 tumors, supporting large-scale molecular profiling. Industry collaborations further highlighted the translational utility of these resources.

**Conclusions:**

This study describes the development and application of a gynecologic cancer biobank that integrates standardized biospecimen collection, rigorous QC, and the generation of diverse secondary resources. By linking these resources with clinical and epidemiological data, the biobank provides a scalable and accessible platform for precision oncology and academic–industry collaboration.

## Introduction

Since 2008, the Korea Biobank Project (KBP), led by the Korea Disease Control and Prevention Agency (KDCA), has established a national infrastructure to support precision medicine and the bio-health industry through the systematic collection and distribution of human biospecimens. To standardize operations and facilitate large-scale data integration, the Korea Biobank Network (KBN) was launched, linking biospecimens with clinical and epidemiological data under a unified Common Data Model (CDM v3.0) [1]. As of 2022, over 660,000 participants have contributed biospecimens to this national effort.

To accelerate translational research and industrial applications, KBP entered its fourth phase (2021–2025) under the KBP Vision 2030, moving beyond conventional disease-based repositories toward investigator-driven projects that actively connect biospecimen collection with utilization. Within this framework, the Innovative Biobanking Consortium Support Program was introduced to provide operational support, funding, and infrastructure integration for disease-focused biobanks. The Innovative Biobanking Consortium Support Program provides centralized support for participating research teams—including funding, operational training, system integration, and regulatory alignment—while allowing decentralized biospecimen collection tailored to specific diseases. Compared to other international gynecologic cancer biobanking initiatives, the KBP is distinguished by its nationwide integrated infrastructure, real-time data connectivity, and disease-focused secondary resource development. These features strategically position KBP as a scalable platform for precision medicine research.

Through this program, our team formed a gynecologic cancer biobanking consortium via the Korean Gynecologic Oncology Group (KGOG), a multi-institutional clinical network comprising seven tertiary hospitals. Specimens collected at each participating institution were transferred to Gangnam Severance Hospital, which served as the central operational hub responsible for quality control (QC) and the development of secondary resources. Final deposition of all primary and secondary biospecimens was made at the National Central Human Resources Bank operated by the KDCA.

We participated in this consortium by collecting gynecologic cancer biospecimens and transferring them to the Korea Biobank, while also developing secondary resources to support translational research. We present an operational model in which researchers act as both resource developers and users, thereby driving translational studies and academic-industrial collaboration [2–4]. We collect blood, tumor tissues, ascites, and urine, and generate advanced research tools such as whole-genome sequencing (WGS) data, immortalized human ovarian surface epithelial (IHOSE) cell lines, patient-derived xenograft (PDX) models, tumor organoids, and tissue microarrays (TMA). All resources are deposited in the National Central Human Resources Bank to ensure broad accessibility.

In this study, we present the resource development strategy and operational framework, describe outcomes from biospecimen collection and utilization, and highlight the role of gynecologic cancer biospecimens in advancing precision oncology and biomedical innovation. Through this work, we aim to provide a scalable operational model that supports future translational and discovery-based research in gynecologic oncology.

## Materials and methods

### Participant enrollment

A total of 294 patients with cervical, endometrial, or ovarian cancer were enrolled through the KGOG network, including Gangnam Severance Hospital, Seoul National University Hospital, Korea University Ansan Hospital, Ulsan University Hospital, Korea University Anam Hospital, Ewha Womans University Seoul Hospital, and Chung-Ang University Hospital. Biospecimens collected between 2012 and June 16, 2024 (prior to approval by the Institutional Review Board (IRB)), were retrospectively obtained through the Human Biobank of Gangnam Severance Hospital. The retrospective cohort comprised 179 patients. Access to retrospective biospecimens and linked clinical data for research purposes was granted in July 2024. Only de-identified samples and associated clinical information were provided, and the investigators had no access to identifiable participant information at any stage of data collection or analysis.

Following IRB approval on June 17, 2024 (No. 3-2024-0151), patients were prospectively recruited, and biospecimens were collected from an additional 115 patients after obtaining written informed consent. The informed consent process included a participant information sheet, biospecimen research consent form, and biospecimen donation agreement. Inclusion criteria were: (1) histologically confirmed gynecologic cancer; (2) availability of biospecimens collected during routine clinical care; and (3) age ≥ 19 years. Exclusion criteria included samples not meeting the quality requirements of the National Biobank of Korea or collected outside protocol-defined conditions. De-identified biospecimens and linked clinical data were securely stored with restricted access.

### Biospecimen collection

Primary biospecimens—including blood, tumor tissue, urine and ascites—were collected according to a standardized protocol. Collected specimens were physically transferred to Gangnam Severance Hospital, which served as the central operational hub. Frozen biospecimens were stored at –80 °C in monitored freezers with continuous temperature logging. Each specimen was labeled with a unique 2D barcode to enable traceability. Preanalytical variables were documented using the Standard PREanalytical Code (SPREC) [5]. Barcode and SPREC metadata were integrated into the biospecimen information management systems for seamless tracking. Containers adhered to specifications of the National Biobank of Korea. Selected biospecimens were processed at Gangnam Severance Hospital to generate secondary resources. Detailed processing conditions are summarized in S1 Table. All biospecimens and metadata were registered and managed using the Human Biobank Information System (HuBIS), an integrated platform operated by the National Biobank of Korea. HuBIS includes modules for barcode generation (HuBIS_Tracker), specimen registration and storage (HuBIS_Sam), and distribution management (HuBIS_Desk).

### QC and performance metrics

QC and performance metrics were implemented in accordance with standardized operating procedures to ensure biospecimen suitability for downstream analyses. Quantitative nucleic acid quality assessment was performed in a purpose-dependent manner, with DNA Integrity Number (DIN) evaluated for frozen tissue samples intended for genomic analyses.

Specimens failing predefined QC criteria were excluded from downstream analyses. Specifically, serum and plasma samples were visually inspected for hemolysis, coagulation, or contamination and excluded at an early stage if deemed unsuitable. Buffy coat samples were evaluated using sample-based cellular QC to assess cell viability and cell counts. Frozen tissue specimens were assessed for nucleic acid quality based on DIN measurements, and FFPE samples were reviewed by pathologists to confirm tumor content and overall tissue suitability. Pre-analytical variables were systematically recorded for all specimens using the SPREC version 3.0, enabling comprehensive documentation and traceability of pre-analytical conditions across participating centers.

### Processing and storage of secondary (derived) resources

#### WGS procedures.

WGS was performed on paired tumor-normal samples from patients with gynecologic cancers. Sequencing was conducted in collaboration with Macrogen Inc. (Seoul, Korea) using Illumina NovaSeq platform and INOCRAS Inc. (Seoul, Korea) using the DNBSEQ-T7 platform. Tumor samples were sequenced at a minimum depth of 60× and matched normal samples at ≥ 30 × . Paired design enabled accurate discrimination between somatic and germline mutations. A secure network-attached storage (NAS) system was used for long-term data storage.

#### Establishment of IHOSE cell lines.

HOSE cells were derived from surgical tissues under IRB approval (No. 3-2023-0326) and immortalized using lentiviral vectors encoding either SV40 T antigen or HPV E6/E7 genes, following established protocols [4]. The resulting immortalized HOSE (IHOSE) cell lines were validated by STR profiling, screened for mycoplasma contamination, and cryopreserved for downstream applications. Full protocols are described in S1 File.

#### Reimplantation and viability assessment of cryopreserved PDX tumor tissues.

All animal experiments were conducted in accordance with institutional guidelines and were approved by the Institutional Animal Care and Use Committee (IACUC) of Yonsei University Health System (Approval No. 2022−0158). Six-week-old BALB/c nude mice were purchased and acclimated to the animal facility for one week prior to the initiation of experiments. Cryopreserved PDX tumor tissues were retrieved from cryostock stored at –80 °C for short- to mid-term storage. Tumor fragments (approximately 0.3 × 0.3 × 0.3 cm) had been preserved in Cell Banker freezing medium and were rapidly thawed in a 37 °C water bath for approximately 90 seconds. Each thawed tumor fragment was subcutaneously implanted into two recipient mice.

For surgical implantation, mice were anesthetized using a combination of alfaxalone (76 mg/kg) and xylazine (10 mg/kg). Approximately 10 minutes after anesthesia induction, a wide surgical area including the incision site was disinfected with povidone-iodine. A small incision was made in the subcutaneous tissue of the shoulder region, and the thawed tumor fragment was inserted into the subcutaneous pocket. The incision was promptly closed using sutures. Mice were continuously monitored until full recovery from anesthesia (approximately 2 hours), and postoperative conditions were carefully observed. Tumor growth was monitored beginning 3–4 weeks after implantation. Tumor length (L; longest diameter) and width (W; shortest perpendicular diameter) were measured weekly using digital calipers, and tumor volume was calculated using the formula V = (L × W^2^)/2. Body weight and clinical signs of distress or discomfort were assessed weekly to minimize animal suffering. Humane endpoints were predefined in the IACUC-approved protocol as tumor diameter ≥2 cm, tumor volume exceeding 5% of normal body weight, ulceration or necrosis of the tumor, or signs of significant distress or impaired physiological function. Animals were euthanized immediately upon reaching any of these criteria. When tumors reached a size sufficient for downstream analyses within the approved humane endpoint limits, mice were euthanized and tumors were harvested.

Harvested xenograft tumors were partially fixed in formalin, paraffin-embedded, and subjected to hematoxylin and eosin (H&E) staining to evaluate histological similarity to the corresponding patient tumors. Regrown PDX tumor tissues were subsequently cryopreserved at –150 °C for long-term storage. At the experimental endpoint, mice were euthanized using a CO₂ chamber, and death was confirmed by cessation of respiration.

#### Criteria for PDX engraftment success/failure and passage designation.

The viability and engraftment success of cryopreserved PDX tumor tissues were evaluated during a follow-up period of up to five months after implantation. Successful engraftment was defined as tumor growth reaching a volume of ≥200 mm^3^ within the observation period. In contrast, cases in which no tumor growth was observed or tumor volume remained below 200 mm^3^ throughout the five-month period were classified as engraftment failure.

The passage number of the cryopreserved tumor tissue used for reimplantation was denoted as, for example, “P4.” Tumors that successfully grew following reimplantation and were subsequently harvested for the next passage were designated as “P5.” For instance, the notation PDX-50-P4(P5) indicates that a P4 tumor tissue was reimplanted and successfully expanded to generate a P5 xenograft.

H&E-stained slides were scanned at a 400 × optical magnification using a ZEISS Axioscan 7 microscope slide scanner to generate whole-slide images. The acquired images were imported into QuPath (version 0.4.4), and representative regions were selected for image capture.

#### Tumor organoids.

Primary ovarian cancer tissues were collected with informed consent and Institutional Review Board approval (No. 3-2023-0111). Organoids were generated by enzymatic digestion, Matrigel embedding, and culture in a defined medium modified from the ATCC ovarian cancer formulation (composition detailed in S2 Table). Histological validation was performed using H&E and immunohistochemistry for pan-cytokeratin (PanCK; mouse monoclonal, clone AE1/AE3 + 5D3, Abcam, cat# ab86734, 1:500) and Ki-67 (rabbit monoclonal, clone SP6, Abcam, cat# ab16667, 1:500). For drug testing, organoids were dissociated into single cells, seeded in 96-well plates, and treated with cisplatin, carboplatin, or nab-paclitaxel across a range of concentrations for 72 hours. Cell viability was measured using the CellTiter-Glo® 3D assay (Promega). Detailed protocols are provided in S1 File.

#### TMA construction.

TMAs were constructed retrospectively using archived formalin-fixed paraffin-embedded (FFPE) tumor tissues obtained from the Korea Gynecologic Cancer Bank. TMA construction was performed using specimens that partially overlapped with the main study cohort. For endometrial cancer, TMAs were generated from tumor tissues of 370 patients collected retrospectively (November 20, 2007–June 7, 2024), with 69 cases overlapping with the main study cohort (n = 294). For cervical cancer, TMAs were constructed from tumor tissues of 149 patients collected (March 4, 2000–October 28, 2024), with 78 cases overlapping with the main cohort.

Representative tumor regions were reviewed and selected by pathologists, and a single core (1.0–1.5 mm in diameter) was taken per case. These tissue cores were punched from donor blocks and arrayed into recipient paraffin blocks. TMA sections were stained with H&E and digitally scanned for evaluation. Core retention, tumor content, and staining quality were systematically assessed, and only cores meeting predefined quality criteria were included for downstream histopathological and biomarker analyses.

### Ethics statement

All procedures involving human participants were conducted in accordance with the Declaration of Helsinki (2013 revision). The study protocol was approved by the IRB (No. 3-2024-0151), and additional approvals were obtained from local IRBs at participating institutions. Animal experiments were approved by the Institutional Animal Care and Use Committee (IACUC) of Yonsei University Health System (Approval No. 2022−0158) and conducted in compliance with institutional and national guidelines, with predefined humane endpoints.

## Results

### Biospecimen and data collection

**Fig 1:**
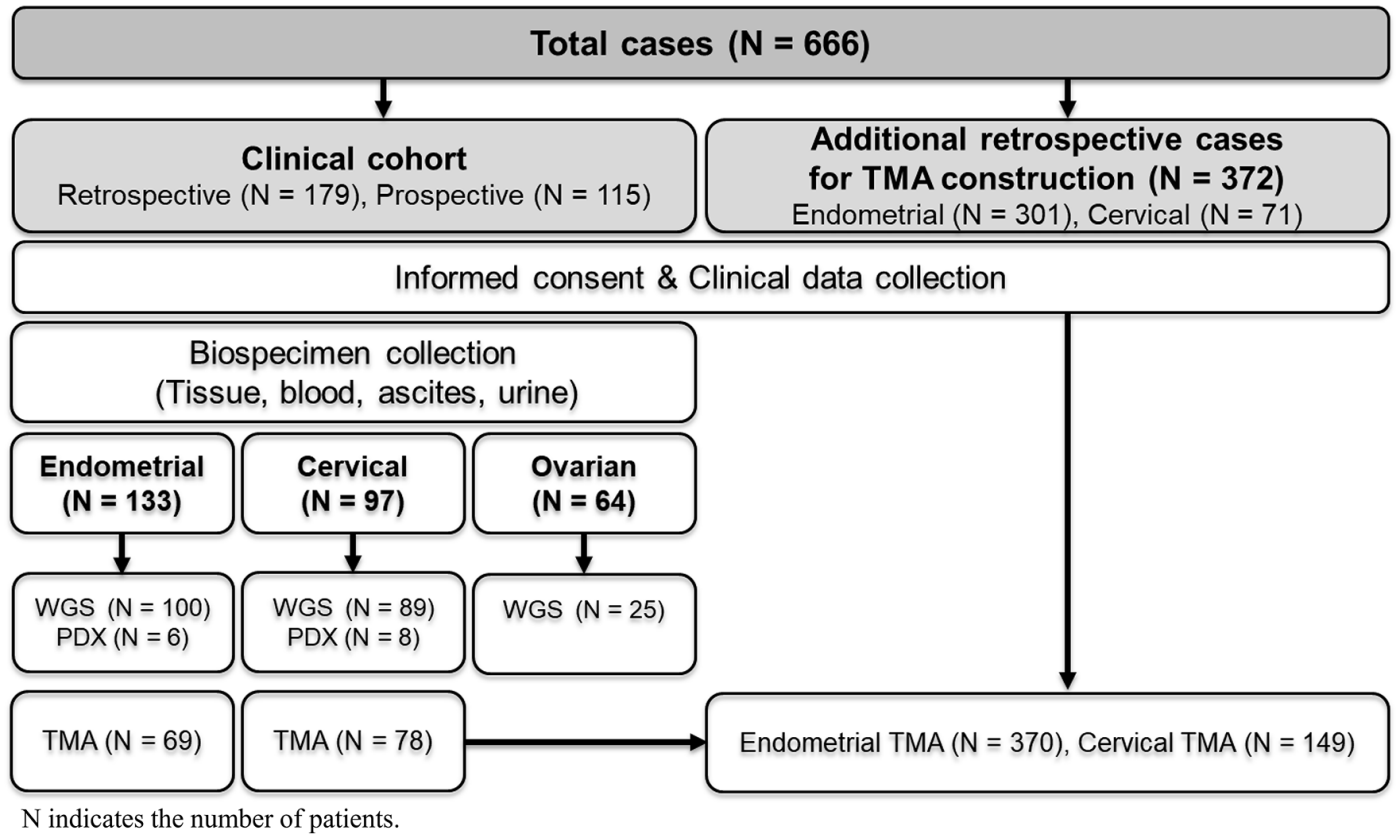
Overview of biospecimen collection and resource development. A total of 666 gynecologic cancer cases were included in the biobank. Among these, 294 cases formed the clinical cohort with informed consent and included retrospective (N = 179) and prospective (N = 115) cases from patients with endometrial, cervical, or ovarian cancers. For each case, biospecimens (tissue, blood, ascites, urine) and clinical data were collected. Derived secondary resources include whole-genome sequencing (WGS) datasets, patient-derived xenografts (PDXs), and tissue microarrays (TMAs). An additional 372 retrospective cases were used for TMA construction (endometrial: N = 301; cervical: N = 71), all under IRB approval and with patient consent. N indicates the number of patients.

An overview of the study design, cohort composition, and biospecimen collection workflow is summarized in Fig 1. A total of 294 patients with gynecologic cancers were enrolled, comprising 133 with endometrial cancer, 97 with cervical cancer, and 64 with ovarian cancer. Clinicopathological characteristics—including patient age, tumor stage, histological subtype, and grade—are summarized in the S3 Table. From these participants, 6,168 biospecimens were collected, including blood (serum, plasma, and buffy coat), tissues (fresh frozen and FFPE), urine, and ascites. Detailed distributions by cancer type and biospecimen are shown in Table 1. All biospecimens were processed and stored according to standardized protocols, with QC procedures implemented using the SPREC. Special attention was given to preserving purity and integrity during collection and processing to ensure suitability for downstream applications.

**Table 1:** Number of biospecimens collected by cancer type.

Disease	Patients	No. of Cases [Vials]						
		Serum	Plasma	Buffy coat	FFT^*^	FFPE	Urine	Ascites
**Endometrial Cancer**	133	133 [665vials]	133 [664vials]	133 [133vials]	133 [504vials]	133 [133vials]	92 [459vials]	23 [115vials]
**Cervical Cancer**	97	97 [485vials]	97 [485vials]	97 [97vials]	97 [359vials]	97 [97vials]	72 [356vials]	34 [166vials]
**Ovarian Cancer**	64	64 [320vials]	64 [320vials]	64 [64vials]	64 [274vials]	64 [64vials]	55 [275vials]	27 [133vials]

*FFT: Fresh Frozen Tissue.

In parallel, clinical and epidemiological data were collected following the CDM v3.0 of the KBN and curated in the KBN BRIDGE system. The dataset included demographic, clinical, laboratory, pathology, and imaging information, summarized in Table 2. These data are linked with biospecimens and made available to researchers through the Korea Biobank, thereby enabling integrated biomedical and translational research.

**Table 2:** Summary of clinical and diagnostic data.

Category	Data Collected
**Clinical Info**	KBN_ID, gender, birthdate, height/weight, medical/surgical history, smoking/alcohol history, ICD code, staging, treatment details, surgery date/type, recurrence and follow-up, survival/death info
**Lab Tests**	CA125, CA19−9, SCC Ag, CEA, CA15−3, gBRCA, HRD, dMMR, tNGS, PD-L1, PD-1
**Pathology**	Histologic subtype, tumor grade, lymphovascular invasion, pathologic stage
**Imaging**	OB ultrasonography, CT abdomen/pelvis, CT chest, PET-CT (torso)

### WGS and paired tumor-normal analysis

WGS was conducted on biospecimens from 386 gynecologic cancer cases, including 172 paired tumor–normal sets. By cancer type, the cohort comprised 181 endometrial, 170 cervical, and 35 ovarian cancers. Paired tumor–normal datasets were available for 81 endometrial, 81 cervical, and 10 ovarian cancer patients, with matched normal DNA derived from either peripheral blood lymphocytes or adjacent non-tumorous tissues. Sequencing was performed using the Illumina NovaSeq platform for endometrial and cervical cancer samples, and the DNBSEQ-T7 platform for ovarian cancer samples. Tumor DNA was sequenced to an average depth of 90× for endometrial and cervical cancers and 60× for ovarian cancers, while all matched normal samples were sequenced at ≥30 × coverage. This design allowed accurate discrimination of somatic and germline mutations, thereby facilitating robust downstream genomic analyses.

### Establishment and validation of IHOSE cell lines

**Fig 2:**
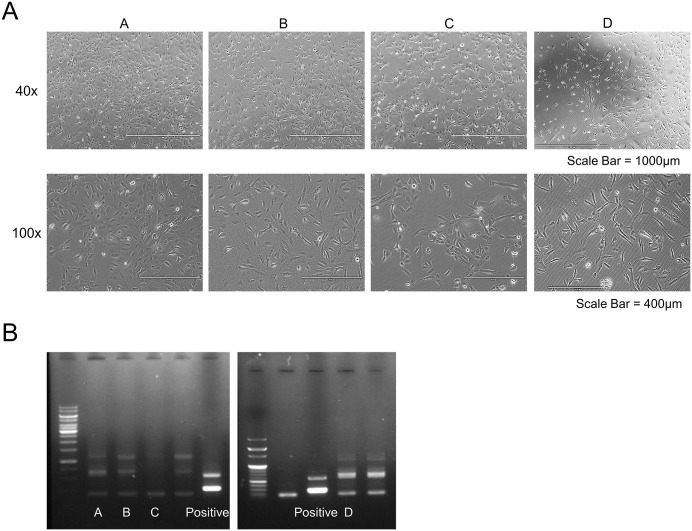
Establishment and validation of IHOSE cell lines. **(A)** Phase-contrast microscopy images of four IHOSE cell lines (designated A–D) at ×40 and ×100 magnification. All cell lines retained epithelial morphology and showed stable proliferative capacity. Scale bar = 1000 µm (×40), 400 µm (×100). **(B)** PCR-based mycoplasma contamination testing. No contamination was detected in any of the four lines. The positive control (+) confirmed the assay sensitivity.

Immortalization was attempted in ovarian surface epithelial cells derived from nine independent normal ovarian samples, resulting in the successful establishment of four IHOSE cell lines (Fig 2A). Although increased proliferative activity was observed immediately after immortalization, some samples exhibited growth arrest during subsequent culture. Therefore, in this study, successful establishment of an IHOSE cell line was defined as sustained and consistent proliferative capacity observed across both early and late passages. Cell line authentication was performed using STR profiling, and all cell lines tested negative for mycoplasma contamination using PCR-based assays (Fig 2B). The established cell lines were cryopreserved, thawed, and re-cultured to confirm post-thaw viability and stability. Only cell lines that passed all QC assessments were deposited into the National Biobank.

### PDX model development and *in vivo* validation

**Fig 3:**
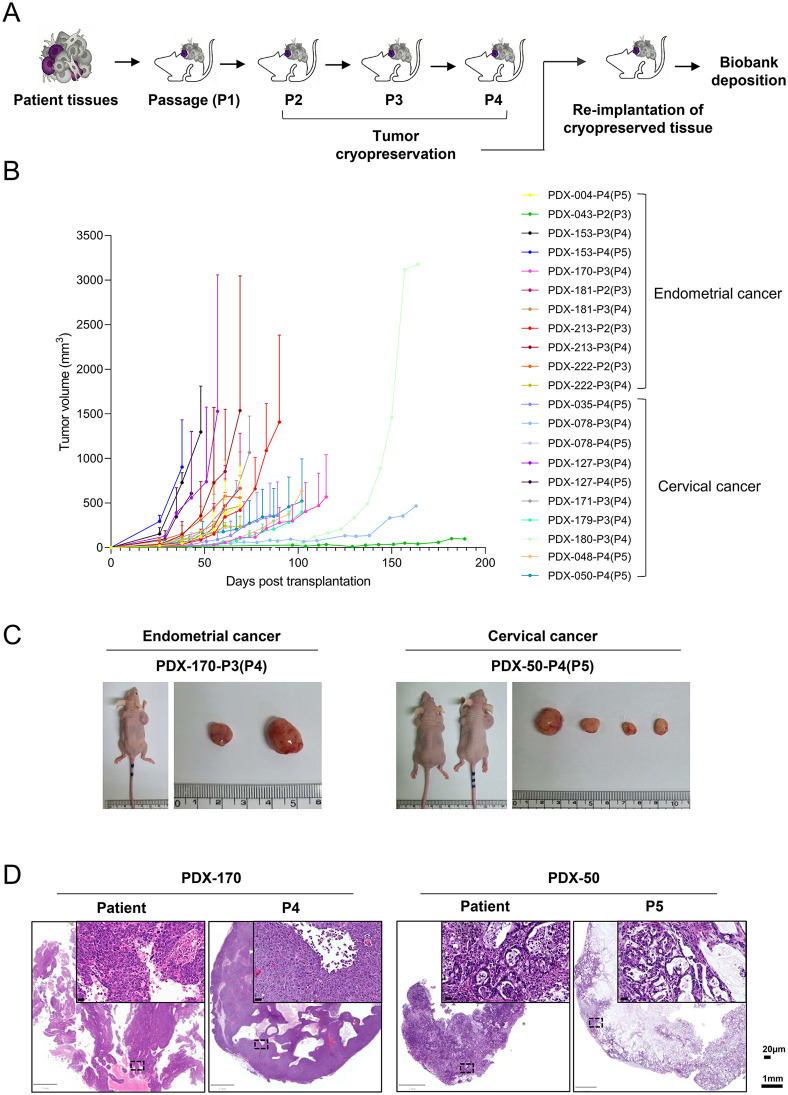
Re-implantation–based validation of cryopreserved PDX tumor tissues for biobank deposition. **(A)** Schematic overview of the study workflow illustrating establishment of PDX models from gynecologic cancer tissues, serial passaging (P1–P4), cryopreservation of tumor tissues, re-implantation of cryopreserved PDX fragments, and subsequent deposition into the biobank. **(B)** Tumor growth curves following re-implantation of cryopreserved PDX tissues derived from endometrial cancer (upper panel) and cervical cancer (lower panel). Tumor volumes were measured weekly using digital calipers and plotted over time. The passage number indicated in parentheses (P#) represents the passage of the cryopreserved PDX tissue used for re-implantation, followed by the passage number of the regrown xenograft. **(C)** Representative gross images of BALB/c nude mice bearing re-implanted PDX tumors (left) and the corresponding harvested xenograft tumors (right). Representative examples from endometrial cancer (PDX-170-P3(P4)) and cervical cancer (PDX-50-P4(P5)) are shown. **(D)** Representative H&E–stained sections of the original patient tumors (left) and the corresponding re-implanted xenograft tumors (right) are shown. Whole-slide images with higher-magnification insets from endometrial cancer (PDX-170, P4) and cervical cancer (PDX-50, P5) demonstrate preservation of histological features following re-implantation.

Our institution has previously established PDX models from gynecologic cancer tissues and cryopreserved the corresponding PDX tumor tissues [6]. Prior to deposition into the National Biobank, cryopreserved PDX tumors were reimplanted to assess their viability, defined as regrowth potential and preservation of histological features. The overall workflow of thawing, reimplantation, monitoring, validation, and biobank deposition is summarized in Fig 3A. Reimplantation was attempted in a total of 19 PDX models, of which 14 exhibited successful tumor regrowth, corresponding to an overall success rate of 73.7% (14/19) (S4 Table). When stratified by cancer type, reimplantation success rates were 72.7% (8/11) for cervical cancer PDX models and 75.0% (6/8) for endometrial cancer PDX models. The passage number (P#) indicates the passage of the cryopreserved PDX tissue used for reimplantation. When available, tumor tissues from different passages of the same model were reimplanted to assess passage-dependent regrowth. In two models (PDX-04 and PDX-170), early-passage cryopreserved tissues failed to regrow tumors, whereas later-passage tissues from the same models resulted in successful regrowth. Although limited in number, these cases indicate that reimplantation outcomes may vary according to the passage stage of the cryopreserved tissue.

Tumor growth was monitored weekly, and representative growth curves, including those from different passage tissues, are shown in Fig 3B. In contrast, PDX-43 did not reach the predefined engraftment threshold of 200 mm^3^ within five months after implantation and showed a maximum tumor volume of 97.7 mm^3^ over a total observation period of 189 days; this model was therefore classified as reimplantation failure. Representative gross images of regrown tumors and host mice are shown in Fig 3C. Histological evaluation using H&E staining confirmed preservation of histological features in regrown PDX tumors (Fig 3D). Collectively, reimplantation-based validation of cryopreserved PDX tumor tissues demonstrated an overall success rate of 73.7%, supporting their suitability for deposition into the National Biobank and subsequent distribution for research use.

### Establishment of patient-derived organoids for drug evaluation

**Fig 4:**
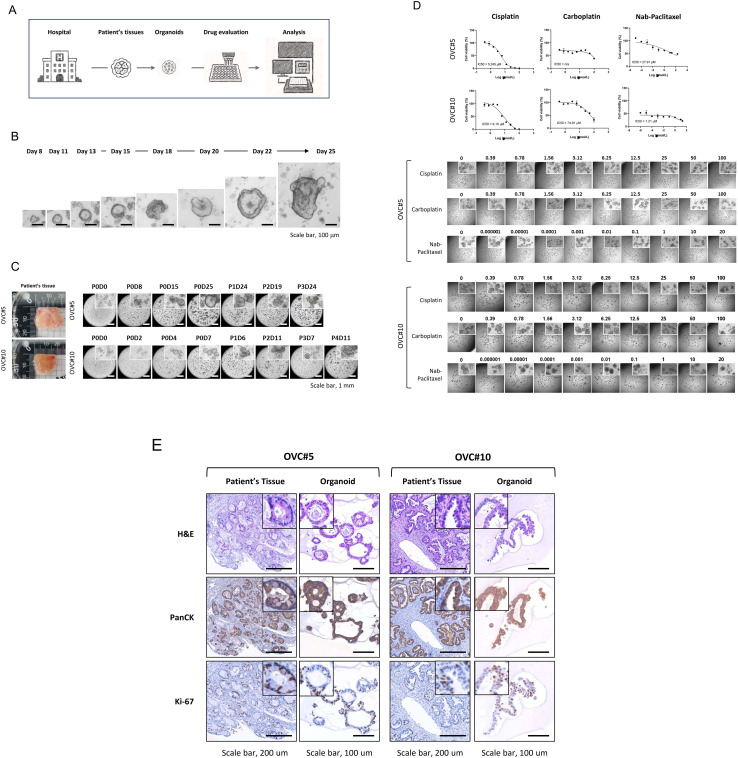
Establishment and characterization of patient-derived ovarian cancer organoids. **(A)** Schematic workflow illustrating the overall experimental process: ovarian cancer patient tissues were collected from the hospital, followed by organoid establishment, drug efficacy testing, and data analysis. **(B)** Brightfield images showing the growth kinetics of patient-derived ovarian cancer organoids (OVC#5) over time. Organoids increased in size and complexity from Day 8 to Day 25. Scale bar = 100 μm. **(C)** Serial passage of organoids derived from two ovarian cancer patients (OVC#5 and OVC#10). Representative brightfield images of organoids at each passage are shown. Scale bar = 1 mm. **(D)** Drug sensitivity assays for cisplatin, carboplatin, and nab-paclitaxel in OVC#5 and OVC#10 organoids. Top: Dose–response curves with calculated IC₅₀ values. Bottom: Representative organoid morphology under increasing drug concentrations. **(E)** Histological comparison between original patient tumor tissues and derived organoids using H&E, pan-cytokeratin (PanCK), and Ki-67 staining. Organoids retained epithelial characteristics and proliferative capacity consistent with their parental tumors. Scale bars = 200 μm (tissue), 100 μm (organoid).

Ovarian tumor tissues were collected from ten patients under IRB-approved protocols (No. 3-2023-0111, Fig 4A). Of these, six were pathologically confirmed as ovarian cancer, and organoid cultures were successfully established from two cases (OVC#5 and OVC#10), resulting in an organoid establishment rate of 33.3% (S5 Table). Tumor tissues were enzymatically dissociated and cultured in Matrigel with defined medium. Over time, both OVC#5 and OVC#10 lines developed stable 3D structures and demonstrated continuous expansion across multiple passages (up to passage 3 for OVC#5 and passage 4 for OVC#10; Figs 4B and 4C), indicating robust growth and culture stability.

Drug sensitivity testing revealed a dose-dependent reduction in cell viability in response to cisplatin, carboplatin, and nab-paclitaxel in both organoid lines (Fig 4D). Notably, these *in vitro* responses were partially concordant with the clinical chemotherapy outcomes: OVC#10, which showed partial response (PR) to platinum-based therapy in the patient, also demonstrated measurable sensitivity in the organoid assay; whereas OVC#5, clinically annotated as non-sensitive, showed limited responsiveness *in vitro* (S5 Table). Histological analysis confirmed that the organoids retained key tumor features observed in the original patient tissues. OVC#5 organoids exhibited glandular tubal-type morphology with low Ki-67 expression, while OVC#10 organoids derived from a tumor with mixed clear cell and endometrioid histology displayed predominant papillary structures and higher proliferative activity. Pan-cytokeratin and Ki-67 immunostaining further supported the preservation of epithelial identity and tumor-specific proliferation patterns (Fig 4E).

### Construction of TMA and clinicopathological characterization

A cervical cancer TMA was constructed using tumor tissues from 149 patients and 10 adjacent normal tissues, organized into two FFPE blocks. The clinicopathological characteristics of this cohort are summarized in Table 3. The median patient age was 49 years (range, 25–86 years). Based on FIGO staging, 46.3% of patients were stage I, 18.8% stage II, 23.5% stage III, 4.7% stage IV, and 6.7% presented with recurrent disease. Tumor grades included well differentiated (9.4%), moderately differentiated (32.2%), poorly differentiated (18.1%), and not documented (40.3%). Histological subtypes were predominantly squamous cell carcinoma (76.5%), followed by adenocarcinoma (13.4%), adenosquamous carcinoma (5.4%), and other types (4.7%). Lymph node metastasis was present in 37.1% of cases, absent in 46.1%, and unknown in 16.9%. Lymphovascular space invasion (LVSI), as documented in the original pathology reports, was identified in 44.1% of patients.

**Table 3:** Clinicopathological characteristics of patients in the cervical cancer TMA cohort.

Characteristics	No.	%
Total	149	100.0
Age, Median (range)	49 (25-86)	
FIGO stage		
I	69	46.3
II	28	18.8
III	35	23.5
IV	7	4.7
R	10	6.7
Grade		
Well	14	9.4
Moderate	48	32.2
Poor	27	18.1
Not mentioned	60	40.3
Histology		
Squamous cell carcinoma	114	76.5
Adenocarcinoma	20	13.4
Adenosquamous carcinoma	8	5.4
Others	7	4.7
Lymph node metastasis		
Yes	33	37.1
No	41	46.1
Unknown	15	16.9
Lymphovascular space invasion		
Yes	64	44.1
No	57	39.3
Unknown	24	16.6

An endometrial cancer TMA was constructed using tumor tissues from 370 patients and 158 cases of endometrial intraepithelial neoplasia (EIN), arranged into three blocks. Table 4 summarizes the clinicopathological characteristics of this cohort. The median patient age was 55 years (range, 17–84), and the median BMI was 24.5 (range, 16.7–54.9). Based on the 2023 FIGO staging system, 59.6% of patients were stage I, 19.7% stage II, 17.8% stage III, and 3.0% stage IV. Tumor grades were well differentiated (42.0%), moderately differentiated (32.8%), and poorly differentiated (25.2%). Histological subtypes were predominantly endometrioid adenocarcinoma (88.1%), followed by serous carcinoma (4.6%), clear cell carcinoma (3.8%), and others (3.5%). LVSI, based on clinicopathological records, was observed in 30.5% of patients.

**Table 4:** Clinicopathological characteristics of patients in the endometrial cancer TMA cohort.

Characteristics	No.	%
Total	370	100.0
Age, Median (range)	55 (17-84)	
BMI, Median (range)	24.5 (16.7-54.9)	
FIGO 2023 stage		
I	218	59.6
II	72	19.7
III	65	17.8
IV	11	3.0
Grade		
Well	155	42.0
Moderate	121	32.8
Poor	93	25.2
Histology		
Endometrioid adenocarcinoma	326	88.1
Serous carcinoma	17	4.6
Clear cell carcinoma	14	3.8
Others	13	3.5
Lymphovascular space invasion		
Yes	113	30.5
No	255	68.9
Unknown	2	0.5

These findings illustrate the clinical and pathological heterogeneity represented in the TMA cohort. TMAs enable the inclusion of up to 150 tissue cores in a single FFPE block, thereby reducing cost, time, and technical variability. Beyond conventional immunohistochemistry, TMAs are increasingly applied to advanced omics platforms such as single-cell sequencing and proteomics, underscoring their utility as a versatile tool for precision oncology research.

### Industry collaborations and translational applications

Several industry–academic collaborations were carried out using clinically derived biospecimens, underscoring the translational value of the biobank in real-world applications.

Organoid Science Inc., Korea’s first organoid-based drug development company, established patient-derived gynecologic tumor organoids from tissues collected at Gangnam Severance Hospital. These models are being utilized as platforms for drug response evaluation and will be deposited in the National Biobank of Korea to ensure broader research accessibility.

**Fig 5:**
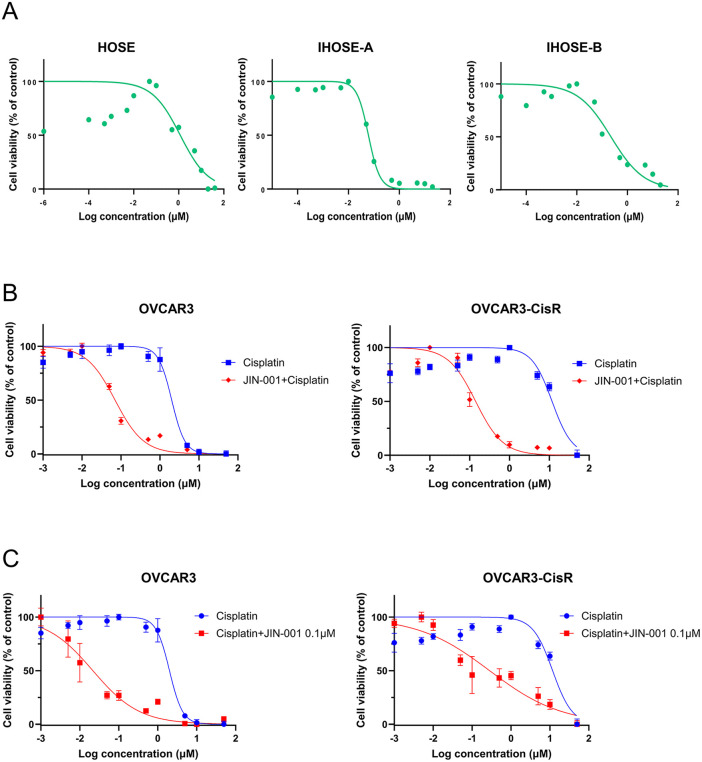
*In vitro* evaluation of JIN-001 in ovarian cancer cell lines. (A) Baseline response profiles of HOSE and IHOSE cells under non-tumorigenic conditions following treatment with JIN-001. (B) Dose–response curves of OVCAR3 and cisplatin-resistant OVCAR3-CisR cells treated with cisplatin in the presence or absence of JIN-001. (C) Cisplatin dose–response curves of OVCAR3 and OVCAR3-CisR cells in the presence of a fixed concentration of JIN-001 (0.1 µM).

J INTS BIO Inc., a biotechnology company specializing in anticancer therapeutics, conducted *in vitro* preclinical studies to evaluate the HSP90 inhibitor JIN-001 using cellular resources accessed through this project. These included HOSE cells and IHOSE cell lines established in this study, as well as a commercially available ovarian cancer cell line (OVCAR3) and a cisplatin-resistant derivative generated from OVCAR3 (OVCAR3-CisR). HOSE cells and two IHOSE cell lines were first used to establish baseline response profiles under non-tumorigenic conditions, prior to evaluation in ovarian cancer cells (Fig 5A). Subsequently, OVCAR3 and OVCAR3-CisR cells were treated with JIN-001 in combination with cisplatin, and increased antiproliferative responses were observed in both cell lines under the tested conditions (Figs 5B and 5C).

INOCRAS Inc., a precision oncology company, applied WGS data generated in this project to its CancerVision^TM^ platform [7]. Ten paired tumor–normal WGS datasets from ovarian cancer patients were used in this study. Homologous recombination deficiency (HRD) status was characterized based on mutational signatures (e.g., SBS3, SBS8, ID6, SV3 and SV5) and the presence of germline BRCA1/2 variants [8], enabling comprehensive genomic profiling of gynecologic tumors.

## Discussion

This study presents our operational framework and resource development strategy for gynecologic cancer biospecimen collection and utilization, and highlights its translational and industrial potential through real-world applications. Biospecimens from 294 patients with endometrial, cervical, and ovarian cancers were collected, and a wide spectrum of high-quality primary specimens (blood, tumor tissue, ascites, urine) and derived resources (WGS data, IHOSE cell lines, PDX models, tumor organoids, and TMAs) were established. Together, these resources constitute a foundational platform for disease-centered precision medicine and illustrate the value of researcher-driven biobank ecosystems.

Among the secondary resources developed, IHOSE cell lines were established and utilized as non-tumorigenic epithelial controls in various studies involving oncogenic pathway analysis, drug sensitivity testing, and biomarker validation [4,9–11]. Notably, immortalized normal epithelial cell lines are widely used across diverse cancer types as essential comparators in translational research [12–14]. These lines have also been requested by multiple external institutions, highlighting their translational relevance and research utility. Similarly, the generation and selection of viable PDX models were informed by accumulated operational data from routine PDX workflows, including tumor growth dynamics, cryopreserved tissue availability, and gross tumor morphology. In line with the biobank’s mission to provide experimentally actionable models, tumors characterized by excessively slow growth or predominantly cystic morphology were deprioritized due to limited experimental value. Instead, models with greater anticipated translational utility were prioritized for reimplantation, and their tumor regrowth consistency and morphological fidelity were assessed using cryopreserved tissues. In parallel with these efforts, patient-derived tumor organoids were also developed and evaluated for their potential as drug testing platforms. Previous work has also demonstrated that patient-derived ovarian cancer organoids can recapitulate tumor histology and pharmacological response patterns, as well as preserve key genomic features of the original tumor, making them useful models for drug sensitivity and resistance testing [15]. Similar to our findings, other studies have reported variability in organoid drug response across patient samples, supporting the potential of organoids to reflect interpatient therapeutic heterogeneity [16]. Nonetheless, some groups using larger cohorts and optimized pipelines have reported higher establishment rates and broader subtype coverage [17], highlighting the need for continued technical refinement. While our sample size was limited, the study confirms that patient-derived ovarian cancer organoids can be reproducibly generated and applied for *ex vivo* drug testing. These models hold promise for a range of translational applications, although further validation with expanded cohorts is warranted to assess their robustness and generalizability.

Importantly, collaborative research with industry partners highlighted the practical impact of biobanked resources. Organoid Science Inc. established patient-derived tumor organoids that provide a promising platform for drug response studies. J INTS BIO Inc. evaluated the anticancer activity of JIN-001, a novel HSP90 inhibitor, and demonstrated synergistic antiproliferative effects when combined with cisplatin, even in resistant cell lines. INOCRAS Inc. leveraged WGS datasets for comprehensive mutational profiling, with focus on HRD characterization in gynecologic cancers. These cases underscore the evolving role of biospecimen collection for gynecologic cancers, functioning not merely as repositories but as dynamic infrastructures that support translational and preclinical research [18,19].

Numerous large-scale biobanking initiatives across the US, UK, Europe, and Japan have pursued complementary goals in gynecologic cancer research. For example, the UK Biobank has enrolled around 500,000 individuals, and the US-led All of Us initiative is targeting over one million participants, with both programs integrating biospecimens, genomic data, and clinical information from diverse populations [20,21]. Japan’s BioBank Japan (BBJ) has amassed samples from approximately 270,000 patients across 51 disease types and added extensive whole-genome sequencing datasets [22]. In Europe, pan-national efforts such as BBMRI-ERIC and dedicated national initiatives—like the Dutch ovarian cancer biobank—facilitate standardized biospecimen collection and multi-institutional collaboration. While these programs emphasize accessibility and scale, they vary in terms of structure, from centralized repositories to distributed consortium-based models. In comparison, the KBP stands out for its nationally integrated infrastructure, linking 43 hospital-based biobanks through a unified governance and informatics framework [23]. KBP places strong emphasis on disease-specific resource generation—including PDX models, organoids, and genomic datasets—coordinated through specialized KBN centers. The system is further strengthened by real-time data integration, automated quality assurance tools, and locally controlled ETL processes that preserve institutional flexibility. These features position KBP as a unique model for translating biobanking infrastructure into scalable precision research platforms.

The established resources are applicable to a wide range of biomedical investigations, extending beyond genomic profiling to include organoid-based drug screening [24], single-cell analysis [25], and spatial omics approaches [26]. Such integration enables comprehensive investigation into tumor heterogeneity, drug responsiveness, and mechanisms of resistance, thereby broadening the research utility of the biobank.

Several limitations should be acknowledged. The relatively small number of ovarian cancer cases (n = 64) was primarily due to retrospective enrollment. During this period, many ovarian cancer biospecimens had already been distributed for previous studies, resulting in a limited number of cases meeting the predefined criteria for integrated biobanking. This imbalance is expected to improve through ongoing prospective specimen collection. Detailed molecular data such as immune phenotyping and transcriptomics are still in progress. In addition, biospecimen collection and the generation of secondary resources are ongoing and are expected to further expand in the coming years. Another limitation is that most patients were enrolled from tertiary referral centers, which may not fully represent the broader population. Furthermore, long-term follow-up data, particularly for treatment response and survival outcomes, are not yet mature but are scheduled for continuous updates as part of the biobank’s operational framework. Future directions include expanding multi-institutional collaboration, increasing the diversity of biospecimen collections, and implementing integrative multi-omics approaches to overcome current limitations and further enhance the role of disease-focused biobanks as core drivers of precision medicine.

## Conclusion

This study demonstrates the establishment of a comprehensive framework for gynecologic cancer biobanking, encompassing harmonized specimen acquisition, validated quality assurance measures, and the creation of advanced research assets. By integrating these components with rich clinical and epidemiological datasets, the biobank offers a robust platform to support secondary resources were developed. Continued biospecimen collection, expansion of secondary resources, and future incorporation of multi-omics analyses will further strengthen the research infrastructure for gynecologic cancers and accelerate biomedical innovation.

## Supporting information

S1 File Supplementary material and methods. (DOCX)

S2 File Original uncropped gel image corresponding to the left panel of Fig 2B. (TIF)

S3 File Original uncropped gel image corresponding to the right panel of Fig 2B. (TIF)

S1 Table Standardized processing and storage protocols applied to various types of biospecimens collected by KGCB. (DOCX)

S2 Table Reagents used for tumor organoid culture. (DOCX)

S3 Table Clinicopathological characteristics of the study cohort (n = 294). (XLSX)

S4 Table Summary of re-implantation outcomes and regrowth characteristics of cryopreserved PDX models. (XLSX)

S5 Table Summary of patient characteristics, treatment, and organoid establishment. (XLSX)
