# Supplementary material for: Biobanking of gynecologic cancer biospecimens: Development, quality control, and translational applications
Source: PLoS One. 2026 Mar 31;21(3):e0345861. doi: 10.1371/journal.pone.0345861 (PMC13037971; doi:10.1371/journal.pone.0345861)
Supplement: S1 File — (DOCX) [file pone.0345861.s001.docx]

**Detailed Protocol for IHOSE Cell Line Establishment**

Human ovarian surface epithelial (HOSE) cells were obtained from residual surgical tissues under IRB approval (IRB No. 3-2023-0326). Ovarian surface epithelial cells were collected by gently brushing the ovarian surface epithelium, and the brushes were immediately immersed in culture medium to recover epithelial cells. The recovered cell suspension was centrifuged and directly seeded onto culture dishes for primary culture. Cells were maintained in Dulbecco’s Modified Eagle Medium (DMEM) supplemented with 10% fetal bovine serum (FBS) and 1% penicillin–streptomycin and were serially passaged under standard culture conditions. When cellular senescence was observed during passaging, immortalization was induced.

Immortalization was performed using lentiviral vectors encoding either SV40 T antigen or HPV E6/E7 genes. Viral particle production and lentiviral transduction of HOSE cells were conducted according to previously described protocols ^4^. Stably transduced clones were selected, and expression of viral genes was confirmed by RT-PCR. Cell line identity was authenticated by short tandem repeat (STR) profiling, and mycoplasma contamination was excluded using a PCR-based detection kit (iNtRON Biotechnology, Seongnam, Korea). Established IHOSE cell lines were cryopreserved using a cryopreservation medium (Cell Banker) and stored at –80 °C until use.

**Patient Tissue Processing and Organoid Culture**

Primary ovarian cancer tissues were obtained from Gangnam Severance Hospital with informed consent under Institutional Review Board approval (IRB No. 3-2023-0111). Immediately after resection, tissues were transported on ice in Advanced DMEM/F12 medium to the laboratory. Upon arrival, tissues were minced into ~3 mm³ fragments and enzymatically digested using a solution of 2.5 mg/mL Collagenase II (Worthington Biochemical Corporation, Lakewood, NJ, USA), 10 μg/mL DNase I (Roche, Basel, Switzerland), and 10 μM Y-27632 (Selleck Chemicals, Houston, TX, USA) for 60 minutes at 37 °C with gentle agitation. The digested material was passed through a 100 μm cell strainer (Corning, Corning, NY, USA), centrifuged at 400 × g for 5 minutes at 4 °C, and the cell pellet was resuspended in cold Advanced DMEM/F12 (Thermo Fisher Scientific, Waltham, MA, USA).

Single cells were mixed 1:1 with Matrigel (Corning, Corning, NY, USA) and plated as 20 μL domes in pre-warmed 48-well plates. After Matrigel solidification at 37 °C for 30 minutes, organoid culture medium was added. The organoid culture medium was modified based on the ATCC ovarian cancer organoid formulation and consisted of Advanced DMEM/F12 (Thermo Fisher Scientific, Waltham, MA, USA) supplemented with 1× Glutamax, 1× HEPES, 1× Penicillin-Streptomycin (all from Thermo Fisher Scientific), 10% R-spondin 1 conditioned medium, 50 ng/mL EGF (PeproTech, Rocky Hill, NJ, USA), 10 ng/mL FGF-10, 10 ng/mL FGF2 (both from PeproTech), 1× B27 (Thermo Fisher Scientific), 10 mM nicotinamide (Sigma-Aldrich, St. Louis, MO, USA), 1.25 mM N-acetylcysteine (Sigma-Aldrich), 1 µM prostaglandin E2 (Tocris, Bristol, UK), 10 µM SB202190 (Selleck Chemicals), and 500 nM A83–01 (Tocris). Medium was refreshed every 2–3 days. Detailed composition is provided in S2 Table.

**Drug Evaluation**

Organoids derived from OVC#5 and OVC#10 were enzymatically dissociated into single cells or small clusters and seeded into Matrigel-coated 96-well plates at a density of 2,000 cells per well. After 5 days of organoid generation, cultures were treated for 72 hours with a range of drug concentrations: cisplatin and carboplatin (both from Selleck Chemicals, Houston, TX, USA; 0.39–100 μM), and nab-paclitaxel (Selleck Chemicals; 1 nM–20 μM).
Cell viability was assessed using the CellTiter-Glo® 3D Luminescent Cell Viability Assay (Promega, Madison, WI, USA), and luminescence was measured using a Cytation 5 imaging reader (BioTek, Winooski, VT, USA). In addition, brightfield images of each well were acquired at the 72-hour endpoint using the same Cytation 5 system to document morphological responses to drug treatment. Viability was normalized to untreated control wells and used to calculate IC₅₀ values.

**Histological and Immunohistochemical Staining**

Patient tumor tissues and derived organoids were fixed in 4% paraformaldehyde (Biosolution, Seoul, Republic of Korea; 24 hours for tissue, 1 hour for organoids) and paraffin-embedded. Sections (4 μm) were stained with hematoxylin and eosin (H&E) using standard protocols.

For immunohistochemistry (IHC), deparaffinized and rehydrated sections underwent heat-induced antigen retrieval in EDTA buffer (pH 9.0) and quenching of endogenous peroxidase activity using 3% hydrogen peroxide (Dako, Carpinteria, CA, USA). Primary antibodies were applied overnight at 4 °C: pan-cytokeratin (PanCK; mouse monoclonal, clone AE1/AE3+5D3, Abcam, Cambridge, UK; #ab86734; 1:500) and Ki-67 (rabbit monoclonal, clone SP6, Abcam; #ab16667; 1:500). After incubation with a biotin-conjugated secondary antibody and streptavidin–HRP complex (ABC kit, Vector Laboratories, Newark, CA, USA), signals were developed using DAB (Vector Laboratories) and counterstained with hematoxylin. Images were captured using the PhenoCycler Fusion system (Akoya Biosciences, Marlborough, MA, USA).
