# Supplementary figures and images for: Biobanking of gynecologic cancer biospecimens: Development, quality control, and translational applications
Source: PLoS One. 2026 Mar 31;21(3):e0345861. doi: 10.1371/journal.pone.0345861 (PMC13037971; doi:10.1371/journal.pone.0345861)

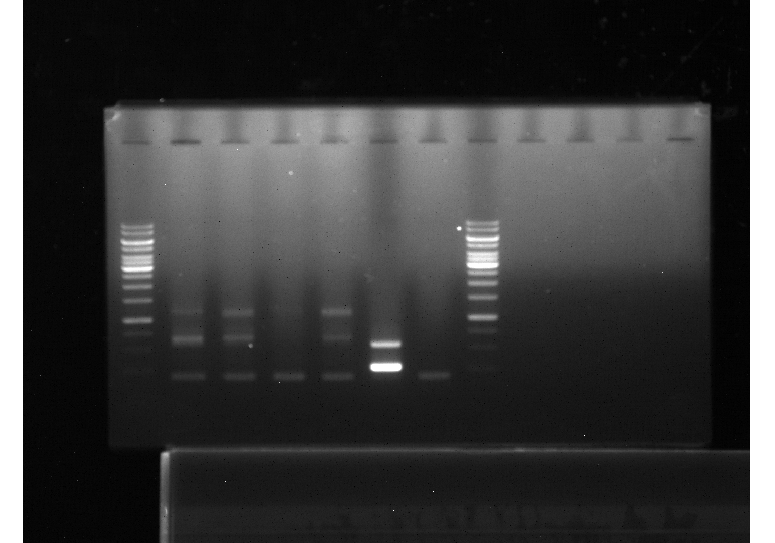

Supplement: S2 File — (TIF) [file pone.0345861.s002.tif]

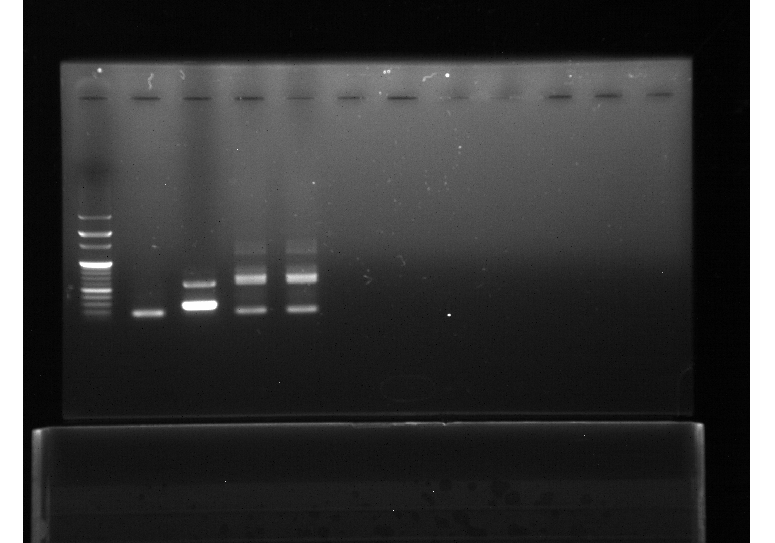

Supplement: S3 File — (TIF) [file pone.0345861.s003.tif]
