# Supplementary material for: Biobanking of gynecologic cancer biospecimens: Development, quality control, and translational applications
Source: PLoS One. 2026 Mar 31;21(3):e0345861. doi: 10.1371/journal.pone.0345861 (PMC13037971; doi:10.1371/journal.pone.0345861)
Supplement: S1 Table — (DOCX) [file pone.0345861.s004.docx]

| **S1 Table. Standardized processing and storage protocols applied to various types of biospecimens collected by KGCB** | | | | | |
| --- | --- | --- | --- | --- | --- |
| Specimen Type | Subtype | Processing Method | Centrifugation | Aliquoting | Storage Temp |
| Blood | Serum | SST tube → centrifuge | 3,000 rpm, 10 min | 300 μL × 5 vials | –80 °C |
|  | Plasma | EDTA tube → centrifuge | 1,800 rpm, 20 min | 300 μL × 5 vials | –80 °C |
|  | PBMC | Density gradient | 1,800 rpm, 5 min | 1×10⁵ cells/vial | –80 °C |
| Tissue | Fresh-frozen | Cut to 0.5 cm cubes | – | 1 cube/vial × 4 | –80 °C |
|  | FFPE | Formalin fixation & paraffin embedding | – | 1 block | Room Temp |
| Urine | Supernatant | Centrifuge | 3,000 rpm, 10 min | 1.5 mL × 3 vials | –80 °C |
|  | Pellet | Mix with RNA later | 3,000 rpm, 10 min | 1 mL × 2 vials | –80 °C |
| Ascites | Supernatant | Centrifuge | 3,000 rpm, 10 min | 1.5 mL × 3 vials | –80 °C |
|  | Pellet | Mix with RNA later | 3,000 rpm, 10 min | 1 mL × 2 vials | –80 °C |
