# Supplementary material for: Biobanking of gynecologic cancer biospecimens: Development, quality control, and translational applications
Source: PLoS One. 2026 Mar 31;21(3):e0345861. doi: 10.1371/journal.pone.0345861 (PMC13037971; doi:10.1371/journal.pone.0345861)
Supplement: S2 Table — (DOCX) [file pone.0345861.s005.docx]

**S2 Table. Reagents used for tumor organoid culture**

| **Reagent /Medium** | **Generic name & description** | **Catalog No.** | **Company, City, Country** |
| --- | --- | --- | --- |
| Collagenase II | Tissue dissociation enzyme (2.5 mg/mL) | C6885 | Sigma-Aldrich, St. Louis, MO, USA |
| DNase I | Endonuclease (10 μg/mL) | DN25 | Sigma-Aldrich, St. Louis, MO, USA |
| Y-27632 | ROCK inhibitor (10 μM) | 1254 | Tocris Bioscience, Bristol, UK |
| Advanced DMEM/F12 | Basal culture medium | 12634-010 | Gibco, Waltham, MA, USA |
| Penicillin–Streptomycin | Antibiotic mixture (100×) | 15140-122 | Gibco, Waltham, MA, USA |
| GlutaMAX | L-alanyl-L-glutamine dipeptide supplement | 35050-061 | Gibco, Waltham, MA, USA |
| HEPES buffer | 1 M solution | 15630-080 | Thermo Fisher Scientific, Waltham, MA, USA |
| R-spondin 1 conditioned media | Growth factor supplement | in-house preparation | Organoid Sciences Inc., Seoul, Korea |
| EGF | Epidermal growth factor  (50 ng/mL) | in-house preparation | Organoid Sciences Inc., Seoul, Korea |
| FGF-10 | Fibroblast growth factor 10  (10 ng/mL) | in-house preparation | Organoid Sciences Inc., Seoul, Korea |
| FGF2 | Fibroblast growth factor 2  (10 ng/mL) | in-house preparation | Organoid Sciences Inc., Seoul, Korea |
| B-27 Supplement (with vitamin) | Serum-free supplement | in-house preparation | Organoid Sciences Inc., Seoul, Korea |
| Nicotinamide | Vitamin B3 derivative (10 mM) | N0636 | Sigma-Aldrich, St. Louis, MO, USA |
| N-acetylcysteine | Antioxidant (1.25 mM) | A7250 | Sigma-Aldrich, St. Louis, MO, USA |
| Prostaglandin E2 | Lipid mediator (1 µM) | BG-0382 | BioGems, Westlake Village, CA, USA |
| SB202190 | p38 MAPK inhibitor (10 µM) | 1264 | Tocris Bioscience, Bristol, UK |
| A83–01 | TGF-β receptor inhibitor (500 nM) | SML0788 | Sigma-Aldrich, St. Louis, MO, USA |
| Matrigel | Basement membrane matrix | 356231 | Corning, NY, USA |
